# Probe Sensor Using Nanostructured Multi-Walled Carbon Nanotube Yarn for Selective and Sensitive Detection of Dopamine

**DOI:** 10.3390/s17040884

**Published:** 2017-04-18

**Authors:** Wed Al-Graiti, Zhilian Yue, Javad Foroughi, Xu-Feng Huang, Gordon Wallace, Ray Baughman, Jun Chen

**Affiliations:** 1 ARC Centre of Excellence for Electromaterials Science, Intelligent Polymer Research Institute, AIIM, University of Wollongong, Wollongong, NSW 2522, Australia; wamag430@uowmail.edu.au (W.A.-G.); zyue@uow.edu.au (Z.Y.); gwallace@uow.edu.au (G.W.); 2 Department of Chemistry, Faculty of Science, Thi-Qar University, Thi-Qar, 64001, Iraq; 3 Centre for Translational Neuroscience, IHMRI, School of Health Sciences, University of Wollongong, Wollongong, NSW 2522, Australia; xhuang@uow.edu.au; 4 The Alan G.MacDiarmid NanoTech Institute and Department of Chemistry, University of Texas at Dallas, Richardson, TX 75083, USA; Ray.Baughman@utdallas.edu

**Keywords:** probe sensor, multi-walled carbon nanotubes, nano yarn, dopamine detection, Nafion coating

## Abstract

The demands for electrochemical sensor materials with high strength and durability in physiological conditions continue to grow and novel approaches are being enabled by the advent of new electromaterials and novel fabrication technologies. Herein, we demonstrate a probe-style electrochemical sensor using highly flexible and conductive multi-walled carbon nanotubes (MWNT) yarns. The MWNT yarn-based sensors can be fabricated onto micro Pt-wire with a controlled diameter varying from 100 to 300 µm, and then further modified with Nafion via a dip-coating approach. The fabricated micro-sized sensors were characterized by electron microscopy, Raman, FTIR, electrical, and electrochemical measurements. For the first time, the MWNT/Nafion yarn-based probe sensors have been assembled and assessed for high-performance dopamine sensing, showing a significant improvement in both sensitivity and selectivity in dopamine detection in presence of ascorbic acid and uric acid. It offers the potential to be further developed as implantable probe sensors.

## 1. Introduction

Dopamine (DA) is a vital neurotransmitter in the central nervous system (CNS). It serves as a chemical messenger between the pre-synapse and post-synapse of adjacent neurons, and plays a critical role in the neurochemical and neurohormonal functions in the mammalian brain [1,2]. Insufficient or excessive level of DA is associated with a number of neurological disorder diseases, such as motricity, Schizophrenia, Alzheimer’s, and Parkinson’s diseases [1,3]. Therefore, DA detection is of great importance in clinical diagnostic applications. Due to the electroactive nature of DA, enormous efforts have been made into electrochemical approaches to develop sensitive and inexpensive devices for rapid detection of dopamine. However, up to now, significant challenges are still present, limiting the efficiency of traditional electrochemical electrodes, in particular for in vivo applications due to their large size (more than 1 mm in diameter) which causes high tissue damage [4,5,6]. For voltammetry detection, most of the stated electrodes have shown a lack of selectivity, with DA signals (oxidation peak) overlapping with uric acid (UA) and ascorbic acid (AA) whose concentrations are typically 10^2^–10^3^ times higher than DA in a biological sample [7,8,9]. Clearly, there is an urgent need for new electrode design and development to address the above challenges.

On the other hand, recent developments in advanced materials are poised to create significant new opportunities for the development of sensors. CNT yarns and sheets already show promising performance for a myriad of applications including electrodes, sensors, supercapacitors, and artificial muscles [10,11,12,13,14]. CNTs can be grown as a forest using a variety of methods, for instance, they can be drawn off and twisted into a CNT yarn for the fabrication of three-dimensional structures. These materials, either in pristine form or in combination with other polymers, metals, or graphene, exhibit superior mechanical, chemical, and electronic properties [12,13,14]. Consequently, they can be potential electrode materials for sensing application. Distinctly, sensors based on CNTs have been proven to be highly sensitive, reproducible, and affordable electrical sensors [15]. The performance of a CNT sensors can be enhanced by functionalization with biocompatible and conductive polymers [16]. For example, surface coating with Nafion (a perfluorinated sulphonated cation exchanger) remains a convenient approach to improve electrode surface characteristics with good selectivity of cations and a long-lasting activated surface [7].

Conventional fabrication methods have shown weakness in meeting the requirements for sensor applications. For example, previous studies have demonstrated the preparation of disk electrodes using CNT yarn for biodetection of DA [17,18]. However, only the tip of the electrodes was used and the lowest concentration could be seen is 1 μM. Also, the experiments did not include AA and UA the common interferences to DA. Therefore, new development on fabrication approaches for probe sensors has emerged as a promising miniaturization method of advanced probe-style bio-electrochemical sensors. With CNT yarns developed in our group, we are able to prepare micro-size probe sensors which could assemble the advantages of basic components [4,12]. First, MWNT fibres can be easily twisted into a yarn with small diameter (i.e., 100–300 µm), which may minimize tissue damage when potentially used for in vivo applications. In addition, CNT fibers have various novel properties, such as high mechanical strength, extraordinary structural flexibility, high thermal and electrical conductivities, corrosion and oxidation resistivity, and high surface area compared to other flexible conducting wires. Moreover, closely packed and uniaxially aligned CNT bundles offer an effective electron pathway for longitudinal current collecting, which makes them ideal for sensors [4,19,20].

To enable optimal sensor performance, carefully selected surface modification can either supply engagement sites of specific molecules or enhance particular chemical reactions as a catalyst [21]. Special surface modification can increase defect density (adsorption sites) and thus maximize the redox reaction of targeted molecules by increasing the electron transfer kinetics [22]. For example, Nafion coating has been extensively used as a simple and attractive surface modification approach for in vivo sensing applications. Due to the negatively charged group (−SO_3_) in its structure, Nafion polymer shows extraordinary permeability to cations, which can enormously facilitate cation loading, resulting in enhanced selectivity and sensitivity towards some vital compounds such as DA. In addition, Nafion coating has been considered one of the long lasting coatings [21,22]. Another advantage of Nafion is that it can be easily applied onto different surfaces via a direct dip coating approach.

In this paper, we demonstrated the use of twisted MWNT yarn fabricated from spinable aligned-MWNT sheets to function as probe sensors, with high selectivity and sensitivity for dopamine detection. We also undertook Nafion coating on the MWNT yarn electrode, and investigated the role of Nafion in obtaining selective detection of DA in presence of UA and AA (common interferences).

## 2. Materials and Methods

### 2.1. Materials

Dopamine hydrochloride (DA, C_8_H_11_NO_2_.HCl), uric acid (UA, C_5_H_4_N_4_O_3_), and ascorbic acid (AA, C_6_H_8_O_6_) were purchased from Sigma-Aldrich. DA, AA, and UA solutions were prepared respectively using freshly prepared PBS buffer. Prior to the electrochemical characterization, all the solutions were purged with nitrogen (N_2_) to remove oxygen. Nafion polymer solution (5%) was purchased from Ion Power, Inc.

### 2.2. Fabrication of the MWNT Yarn

**Figure 1:**
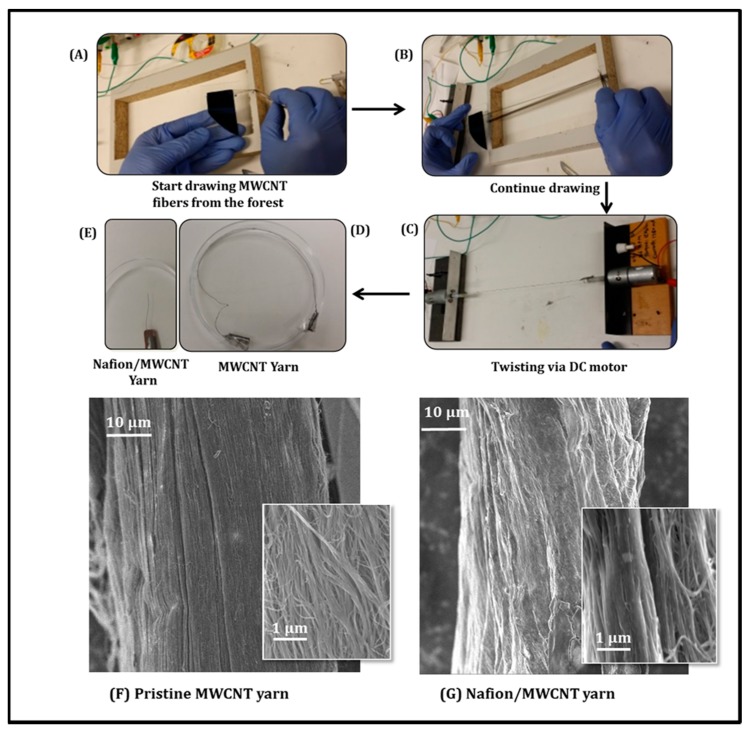
Schematic illustration of the preparation method of Nafion/MWNT Yarn (**A**–**E**); SEM images of MWNT yarn without/ with Nafion coating (**F**,**G**).

The MWNT forest (~400 µm high) for fabricating MWNT yarn was grown by chemical vapor deposition (CVD) using acetylene gas as the carbon precursor as described previously [14,23]. As shown in Figure 1, to prepare the twisted MWNT yarn, arrays of vertically aligned MWNT from the forest are converted into long MWNT sheets by drawing (Figure 1A). The MWNT sheets were attached to both sides of a wooden frame (Figure 1B). Then two ends of MWNT sheets were attached to a DC motor to transform them into yarn filaments by twisting. In addition, a platinum wire (Ø = 20 µm) was twisted into yarn structure during yarn fabrication process (Figure 1C,D). Then, the obtained MWNT yarn electrode was directly dipped into a Nafion solution for 10 min followed with a drying step in an oven at 60 °C. Prior to the Nafion coating procedure, the MWNT yarns were firstly activated by oxygen plasma treatment, which is critical in achieving a uniform and stable Nafion coating on the MWNT yarns.

### 2.3. Physiochemical Characterization of the Microsized MWNT Nanoyarn Probe

The microsized MWNT nanoyarn was tested by Raman spectroscopy using a Jobin-Yvon Horbia 800 (Horiba Jobin Yvon, Edison, NJ, USA), and Fourier transform infrared spectroscopy using the Shimadzu AIM8000 FT-IR spectrometer (Shimadzu Corporation, Kyoto, Japan). The surface morphology of the pristine (Figure 1F) and Nafion-coated (Figure 1G) MWNT yarn was evaluated using a field emission scanning electron microscope, FESEM JEOL7500FA (JEOL Ltd., Tokyo, Japan). It clearly shows that the MWNT yarn was uniformly covered by a Nafion layer after proper dip-coating process [16].

### 2.4. Electrochemical Characterization

The three electrodes system was used for electrochemical performance evaluation including: MWNT yarn as working electrode (WE), Ag/AgCl as reference electrode (RE) and platinum mesh was counter electrode (CE). PBS (at pH = 7.4) was the basic electrolyte in all experiments. The high and low potential (E) were set on 0.6 V and –0.1 V respectively in Cyclic Voltammetry CV testing with applied scan rates ranged from 0.01 to 0.2 (V/s). For Differential Pulse Voltammetry DPV detection, the parameters were as follow: Incr. E = 0.004, amplitude = 0.05, pulse width = 0.05 and pulse period = 0.2. Both CV and DPV were conducted using a CHI-Instrument (660D) electrochemical workstation.

## 3. Results and Discussion

### 3.1. Physiochemical Characterization of the Microsized MWNT Nanoyarn Probe

**Figure 2:**
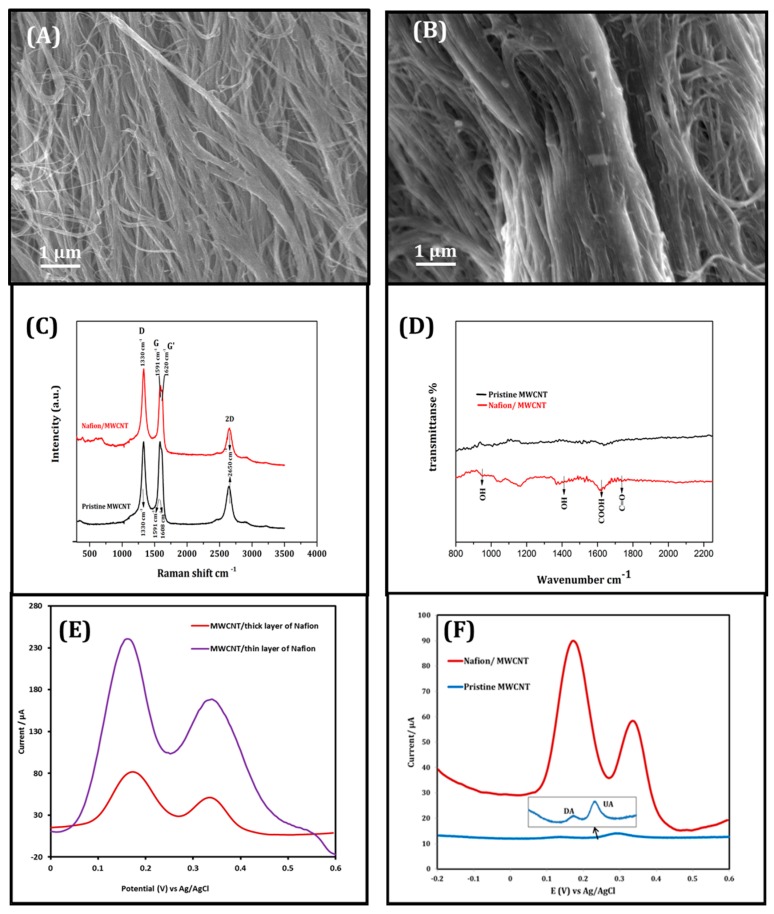
(**A,B**) High resolution SEM images of MWNT bundles without/ with Nafion coating respectively; (**C**) Raman spectra of pristine and Nafion-coated MWNT yarn; (**D**) FTIR spectra of uncoated and Nafion-coated MWNT yarn; (**E**) DPV of different thickness of Nafion layer (10 μM DA with 200 μM UA & AA ); (**F**) DPV of MWNT without and with Nafion coating (10 μM DA + 200 μM AA & UA each).

SEM images (Figure 2A,B) of pristine and modified MWNT yarns showed that the Nafion layer has been successfully coated onto the bundled MWNTs. While the Nafion/MWNT yarn revealed more densely-packed filaments in comparison with the pristine MWNT electrode, due to the occupation of the porous structures among MWNT bundles.

Figure 2C shows the Raman spectra of both pristine and Nafion-coated MWNT yarns. Distinguished *D* and *G* bands are highlighted in the Raman spectra. *D* band relates to the presence of any disorder and functional groups on MWNT walls, and *G* band generates from heterogeneous tubes and carbon bond vibration within graphitic layer [24,25]. Both samples displayed sharp and intense D and G peaks at 1330 cm^−1^ and 1591 cm^−1^ respectively (Figure 2C). Additional resonance 2D at 2650 cm^−1^ (overtone peak of the D band) corresponded to the multiwall structure specifically [26,27]. Furthermore, the Raman of Nafion/MWNT displays a clear increase in D band response, which could be attributed to the Nafion layer related to highly defective structure. −***I_D_/I_G_*** ratio (graphitisation index = *R*) has increased from 1 (the pristine) to 1.11 (the Nafion/MWNT), which supports the increased number of surface defects as well [24]. Moreover, the FTIR spectra (Figure 2D) also show the significant improvement in specific vibrations for OH at 954 cm^−1^, 1412 cm^−1^, COOH at 1616 cm^−1^ and 1707 cm^−1^ for C=O, confirming the presence of a Nafion coating/layer.

### 3.2. Electrochemical Performance of Nafion/MWNT Probe

Preliminary biosensing studies were shown in Figure 2F, to investigate the electrochemical DA detection in the presence of AA and UA (200 μM each), using MWNT probe electrodes with and without Nafion modification respectively. Using the pristine yarn (blue line) only gave minor current signal response of less than 0.5 μA. While Nafion-coated MWNT yarn (red line) displayed a dramatic increase in current signal with highly separated peaks of DA and UA. This improved biosensing performance could be attributed to negatively charged Nafion coating on MWNT yarn. In other words, negatively charged anions on the MWNT skeleton interact with DA cations by electrostatic interactions, which enhance the sensitivity and selectivity towards DA detection in the presence of AA and UA. Further investigations also demonstrate that the thickness of Nafion layer also plays an important role in improving the electrochemical performance. As shown in Figure 2E, a huge improvement in DA sensitivity was observed using thin Nafion layer (purple line) MWNT yarns. This is consistent with the mechanism that thin layer of Nafion could also benefit the faster charge-transfer between Nafion and MWNTs [25].

**Figure 3:**
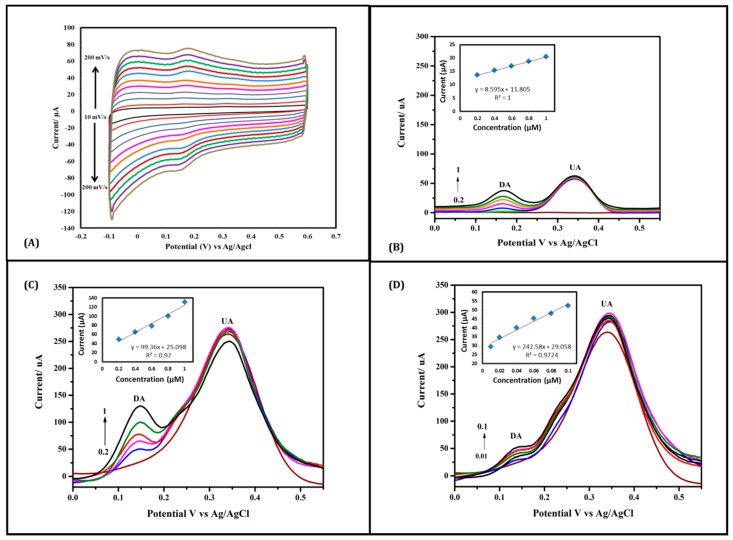
Electrochemical investigation of Nafion-coated MWNT yarn (**A**) CVs of 10 μM DA with 200 μM AA & UA (each) at scan rates: 10, 20, 40, 60, 80, 100, 120, 140, 160, 180, and 200 mV/s; (**B**) DPV of thick layer Nafion in 200 μM UA & AA each with a range of DA: (0.2, 0.4, 0.6, 0.8, and 1) μM in PBS electrolyte with Calibration curve plot between DA concentrations and current response; (**C**) DPV of thin layer Nafion in 200 μM UA & AA with DA range: (0.2, 0.4, 0.6, 0.8, and 1) μM in PBS electrolyte with the calibration curve plot between DA concentrations and current responses; (**D**) DPV of thin layer Nafion in 200 μM UA & AA with a range of DA: (0.01, 0.02, 0.04, 0.06, 0.08, and 0.1) μM in PBS electrolyte with the calibration curve plot between DA concentrations and current response.

Decent electrochemical performance evaluation of thin-layer Nafion/MWNT probe electrode was firstly employed using CV recorded at various scan rates in PBS containing 10 μM DA and 200 μM AA and UA, at low scan rates, both DA and UA have shown well distinguished oxidation peaks at 0.16 V and 0.3 V (vs. Ag/AgCl) respectively (Figure 3). With the increase of scan rate (up to 200 mV/s), DA exhibited a proportional and identical redox couple with a symmetrical shape; however, UA peaks have been clearly depleted with no AA signal being observed throughout the test. Figure 3 demonstrates the significant sensitivity recorded by thin Nafion layer coated MWNT yarn towards DA.

For more detailed investigations, the capability of Nafion-coated MWNT yarn in detecting low concentrations of DA has been tested using DPV as precise and highly sensitive electrochemical method. As shown in Figure 3B–D, DPV curves record the target oxidation of DA (0.0 to 1.0 μM) in a PBS solution containing UA and AA (200 μM each). Typically, DPV curve of Nafion/MWNT yarn showed two well-separated peaks corresponding to DA and UA at 163.95 mV and 335.85 mV, respectively. The current responses increase by increasing its concentration. DA oxidation was notable even at a concentration as low as 0.02 μM, in the presence of 200 μM AA and 200 μM UA. This could be attributed to the surface Nafion coating (high-level negative functional groups) and its selective electrostatic interactions with the amine group (positively charged group) of DA.

In the comparison between thick Nafion layer (dip-coating from concentrated Nafion solution 5%) (Figure 3B) and thin Nafion layer (dip-coating from diluted Nafion solution 0.5%) (Figure 3C), superior DA oxidation signals were observed at the thin-layer Nafion/MWNT yarn electrode. For example, for 0.2 μM DA, the thin Nafion electrode revealed a significant oxidation peak current response around 50 μA, which is 20-times higher than that obtained at the thick Nafion layer electrode. Furthermore, the detection of submicromolar concentrations of DA (as low as 0.01 μM) was also achieved using the thin-layer Nafion/MWNT yarn electrode (Figure 3D). The thin Nafion layer coated MWNT yarn can even detect DA at a low concentration of 0.01 μM with the co-existence of AA and UA (Figure 3D).

**Figure 4:**
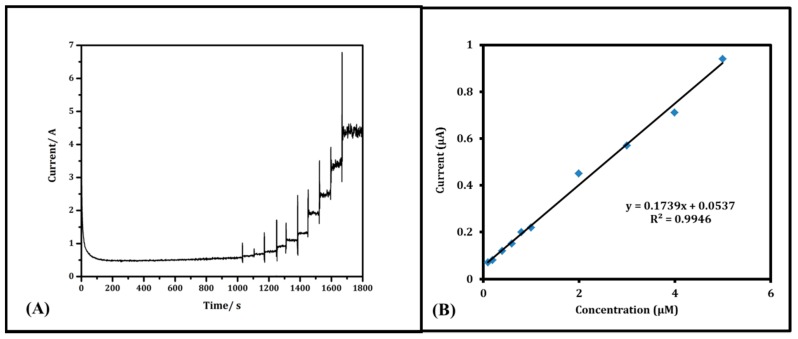
(**A**) Amperometric test of DA (0.1, 0.2, 0.4, 0.6, 0.8, 1, 2, 3, 4, and 5) µM in the presence of 10 µM of AA & UA each; (**B**) Calibration curve of corresponding DA concentrations plot vs. current response.

Figure 4A elucidates the amperometric method to investigate the effect of stated interferences (UA & AA) on DA signals at 0.25 V (vs. Ag/AgCl) resulting from increasing DA concentration (successive additions) from 0.1 to 5 µM with gentle stirring all the time. The current of Nafion/MWNT yarn electrode reached a stable step immediately after each DA addition to the PBS solution. It shows that the presence of UA and AA did not interfere with the DA oxidation. This could be attributed to the effective and efficient catalytic property of the prepared yarn electrode and super-selectivity of Nafion-coating to the DA oxidation at specific voltage [24]. DA oxidation current signals were normalized in Figure 4B, showing a linear relationship between DA concentrations and oxidation current responses. The linear equation is present as I_pa_ = 0.1739 C_DA_ + 0.0537 (R^2^ = 0.9946) in PBS with 200 µM AA and UA. This suggests that DA measurement is reliable using amperometry determination with a Nafion/MWNT probe electrode.

## 4. Conclusions

In summary, our 3D probe structure sensor has shown significant advantages compared to existing literature. Nafion-coated MWNT yarn showed cost effective and rapid electrochemical detection of DA in the presence of UA and AA which confirms the probe-style sensors using CNT yarn. With this electrode, we are able to detect lower concentrations of DA than have already been used in previous works. The electrode was prepared by drawing and twisting CNT forests into micro-sized yarns, and characterized for DA detection using CV and DPV. Indicative anodic peaks of DA and UA were presented at 0.16 V and 0.33 V respectively; while no AA peak was observed, showing excellent selectivity towards DA. In addition, a DA detection limit as low as 0.01 µM was achieved. The as-prepared Nafion/MWNT yarn has demonstrated excellent sensitivity and selectivity of DA detection in the presence of AA and UA. This can be considered a promising probe sensor in DA detection with the potential to be further developed/ integrated into probe sensing devices for clinical applications in the future.
